# A Study of Recombinant Factor IX in Drosophila Insect S2 Cell Lines Through Transient Gene Expression Technology

**Published:** 2018

**Authors:** Jafar Vatandoost, Kambiz Kafi Sani

**Affiliations:** 1. Department of Biology, Hakim Sabzevari University, Sabzevar, Iran; 2. Department of Biotechnology, Sabzevar Branch, Islamic Azad University, Sabzevar, Iran

**Keywords:** *Drosophila* S2 cell, Factor IX, Transient gene expression

## Abstract

**Background:**

Since the mass production of recombinant proteins requires the development of stable cell lines which is a time-consuming complex process, the use of transient expression on a large scale can be a comparatively useful alternative. Although various cell lines have been used for the expression of recombinant proteins, only a limited number of cells enjoy a high transfection characteristic and the ability to adapt to serum-free suspension culture easily. In the present study, the S2 cells from Drosophila insect with the ability to grow in suspension and serum-free cultures were used for the expression of factor IX (FIX) using Transient Gene Expression (TGE) technique.

**Methods:**

*Drosophila Schneider* (S2) cells were seeded in special roller bottles, and then, the cells were transfected with pMT-hFIX plasmid employing the calcium phosphate co-precipitation method. The stable S2-hFIX cells were also seeded in special roller bottles, separately. After the induction, recombinant FIX was quantified in conditioned media employing an ELISA. Moreover, its functional activity was examined using an aPTT assay.

**Results:**

The results showed that the expression of FIX through TGE technology was 1.6 times as high as that obtained through S2-FIX stable cells. Furthermore, the comparison of the FIX expression in S2 cells through TGE techniques with that obtained in previous studies in HEK cells or CHO cells revealed that S2 cells were more efficient in terms of FIX expression.

**Conclusion:**

The S2 cells with the capability to grow in suspension and serum-free cultures are a suitable alternative for transient expression for the large scale production of proteins.

## Introduction

The prerequisite step for the mass production of proteins is to develop stable clones from recombinant cells. Because the production of stable clones using cells is a time-consuming complex process, using the Transient Gene Expression (TGE) technique can be of great significance. With the vast developments made in the transferring methods and transient culturing methods, the mass production of recombinant proteins through transient transferring methods seems viable. In fact, implementing faster methods in the production of recombinant proteins is necessary for the study and comparison of different suitable proteins and their variants in terms of their biological features and characteristics. To this end, the TGE technique seems a comparatively more suitable alternative. Compared to Stable Gene Expression (SGE), TGE allows recombinant proteins to be produced in a relatively shorter period of time (usually in 10 days) in the absence of genetic selection of plasmid DNA 1–3.

Although a large number of cell lines have been used for the expression of recombinant proteins, only a limited number of them enjoy the necessary characteristics listed below: 1) a high transfection characteristic through the usual methods, 2) an ability to adapt to suspension cultures in serum-free cultures, and 3) cost-effectiveness 4.

Mammalian expression systems like CHO and HEK cells are implemented for the production of human recombinant proteins 4. However, using cells which can easily grow in serum-free cultures is cost effective in that serum is expensive to prepare and its use is usually associated with many complications in the purification process 5. Moreover, this technique is used for cells which show a better growth in suspension cultures than they do in plates 4.

The S2 cell line from Drosophila insect, capable of growth in suspension cultures and serum-free cultures, is a suitable expression system for the production of recombinant proteins using TGE technique 5. The S2 expression system has a number of advantages such as a good growth with a high density, and the ability to grow at room temperature in the absence of CO_2_ 6. Hence, the present study aimed at using the S2 cell lines for the factor IX (FIX) expression through TGE technique, and comparing the results with those obtained through stable expressions.

## Materials and Methods

*Drosophila Schneider* (S2) cells were maintained at 28*°C* in the absence of CO_2_ under normal atmosphere in Schneider’s insect medium (Sigma-Aldrich) supplemented with penicillin G (50 *units/ml*) and streptomycin (50 *μg/ml*) (Sigma-Aldrich). One day prior to transfection, 10^9^ cells were seeded in a volume of 300 *ml* in special roller bottles (Sigma), upon which the cells were allowed to loosely adhere. The S2 cells were transfected with pMT-hFIX plasmid employing the calcium phosphate co-precipitation method with minor modifications 7. Cell density and viability were monitored prior to and following transfection by trypan blue exclusion using a 0.4% (*w/v*) solution.

For the assessment of FIX expression in stable clones, the stable S2-hFIX cells, previously described 8, were also seeded in special roller bottles with 10^9^ cell/300 *ml* density. In both transient and stable methods, the FIX expression was screened for 5 days after induction with 0.5 *mM* CuSO4 9 in the presence of 6 *μg* vitamin K1/*ml* 10,11.

Human FIX was quantified in conditioned media employing an ELISA (AsserachromIX: Ag, Stago, Switzerland) according to the procedure provided by the manufacturer. Moreover, the functional activity of recombinant hFIX was examined using an aPTT assay as described before 11. The activity of expressed hFIX was calculated against the standard curve related to normal human plasma (Iranian blood transfusion organization), with one unit of FIX activity corresponding to the amount of FIX in 1 *ml* of normal plasma (∼ 5 *μg/ml*).

## Results

**Figure 1.**
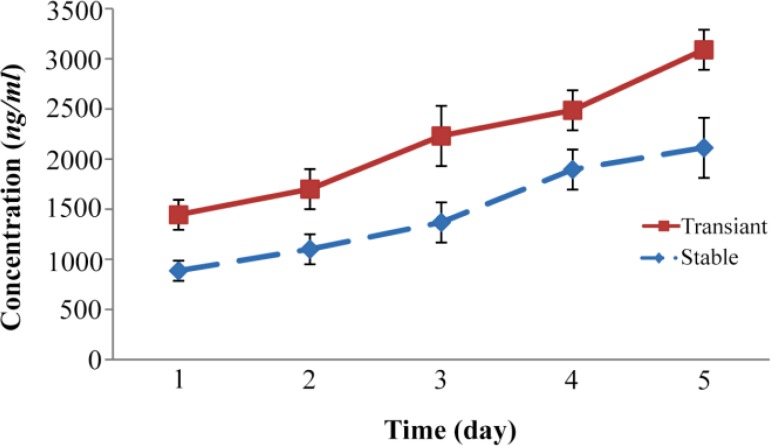
Evaluation of FIX expression in stable S2-FIX cells and transfected Drosophila S2 cells by TGE technique. Following transfection of S2 cells with PMT-FIX constructs, and seeding of stable S2-FIX cells in the roller bottles, FIX expression was induced with 0.5 *mM* CuSO_4_ in the presence of 6 *μg/ml* vitamin K1. Expression at various post induction times was assessed in the conditioned media by ELISA. The data are the means±S.D. of 3 similar experiments.

**Figure 2.**
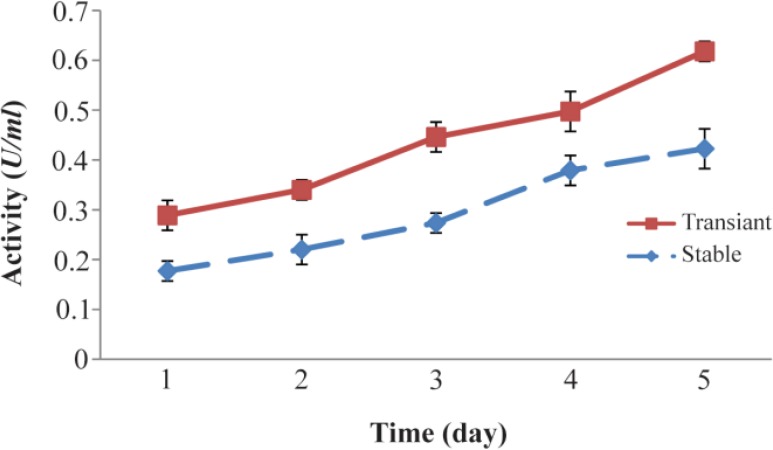
Studying FIX activity in stable S2-FIX cells and transfected Drosophila S2 cells by TGE technique. Following induction of transient or stable S2 cells with 0.5 *mM* CuSO_4_ in the presence of 6 *μg/ml* vitamin K1, at various post induction times, the hFIX coagulation activity of the cultured media was examined by performing clotting test, using immunodepleted plasma for the hFIX and aPTT reagent. The data are the means±S.D. of 3 similar experiments.

The results obtained related to the expression of FIX in the culture of stable S2-FIX cells showed that the amount of FIX produced in five consecutive days were 886, 1101, 1368, 1895, and 2113 *ng/ml*, respectively (Figure 1), indicating an increase in the amount of FIX produced with time. Moreover, the examination of the coagulation time revealed a decreasing trend and when was converted into coagulation activity, it was observed that the coagulation activity increased, with the coagulation activity on the first, second, third, fourth, and fifth days being 0.18, 0.22, 0.27, 0.38, and 0.42 *U/ml*, respectively (Figure 2).

In transient state, after the transfection and the induction of S2 cells in the culture roller bottles, the expression of FIX produced on the first, second, third, fourth, and fifth days were 1445, 1700, 2231, 2487, 3090 *ng/ml*, respectively (Figure 1). Moreover, the comparison of the coagulation activity of the recombinant FIX secreted from transient cells over five days revealed a slight increase in coagulation activity. More specifically, the coagulation activity was measured to be 0.29, 0.34, 0.45, 0.50, and 0. 62, on the first, second, third, fourth, and fifth days, respectively (Figure 2).

The comparison of the FIX expression through stable cells with that through TGE revealed that the expression of FIX through TGE over five days was 1.6 times as high as that produced by stable expression through S2 cells on the same days.

## Discussion

The results obtained in a large number of studies show that TGE technology can be used for the fast, large scale production of recombinant proteins without going through the complex and time-consuming process associated with the production of stable clones 12,13. For the expression of recombinant proteins by TGE technique, usually cell lines are used which grow better in suspension cultures than in plates. From among all mammalian cell lines, HEK293 cells are the most frequently used ones for a large scale transient expression. The expression of human IgG in HEK293 cells as the first successful large scale TGE resulted in the production of 0.5 gram of IgG in less than ten days 14. Recently, a few research groups reported the successful TGE use in CHO cells which had already adopted to suspension conditions 4. As the serum is very expensive to use in cell culture, and is associated with additional complications in purification process, Derouazi *et al* offered a large scale TGE method for CHO cells in the absence of serum 15.

Although CHO cells seem to be the first candidate for the expression of recombinant proteins, S2 cells enjoy considerable advantages such as growth in suspension, and no need for serum, making them a good replacement for the large scale fast production of recombinant proteins 5. This was confirmed by previous studies which showed that the amount of recombinant active FIX was higher in S2 cells than that in CHO cells 8,11. Hence, in this study, the possible use of S2 cell lines with the ability to grow in serum-free suspension cultures for the expression of FIX through TGE technique was examined.

The comparison of the expression of FIX from stable cells (S2-FIX) on a large scale with the expression of FIX through TGE technology showed that the expression of FIX through TGE is higher than that through stable system, being about 1.6 times more than that through stable expression from S2 cells. Although the exact reason for this difference is not well under-stood, the following explanation may be relevant. When the expression construct is inside the S2 cell in both transient and stable states, hundreds of copies of the expression plasmid will spontaneously integrate into the genome in a head to tail fashion 9. However, in expression medium without a selected antibiotic, plasmid stability is observed to be less in stable state. Moreover, cells in the transient state are fresh compared to those in the stable state in that cells need to pass through the selective medium several times in order to acquire the stable status. The counting performed after the induction showed that the viability and the cell density of the stable cells were lower than those of the transient cells.

Similarly, the comparison of the expression of FIX in S2 cells through TGE with that in HEK 16 or CHO cells 17 showed that S2 cells are more efficient.

## Conclusion

In conclusion, considering the aim of the present research study, the TGE technology is one of the best modern up-to-date methods for the large scale fast expression of recombinant proteins, and thus it can replace the current methods in that it can be implemented to produce large amounts of protein with comparatively lower costs. Although this method has not been used so far for a large scale expression of coagulation factors, TGE method has been successfully used in the production of other proteins such as TNF 18, IgG 14, t-PA 13, antibodies 15, secretory, membrane and intracellular proteins 1–3. In all the studies implementing this method, it was emphasized that there are some necessary conditions for the cost-effective large production of recombinant proteins within few days to be possible. Davami showed that TGE is a common method for the expression of recombinant proteins in a short period of time 13. In the same vein, Baldi *et al* showed the feasibility of the use of TGE technology in high and cost-effective production of recombinant proteins using transient transfection of 20 proteins 12. It must be mentioned that all these studies were performed on mammalian cells. Therefore, due to their ability to grow in serum-free suspension cultures, S2 cells can be used in the large scale production of recombinant proteins through TGE technology.
